# Revealing martensitic transformation and α/β interface evolution in electron beam melting three-dimensional-printed Ti-6Al-4V

**DOI:** 10.1038/srep26039

**Published:** 2016-05-17

**Authors:** Xipeng Tan, Yihong Kok, Wei Quan Toh, Yu Jun Tan, Marion Descoins, Dominique Mangelinck, Shu Beng Tor, Kah Fai Leong, Chee Kai Chua

**Affiliations:** 1 Singapore Centre for 3D Printing, School of Mechanical & Aerospace Engineering, Nanyang Technological University, 50 Nanyang Avenue, Singapore 639798; 2 IM2NP, UMR 7334 CNRS, Université Aix-Marseille, 13397 Marseille Cedex 20, France

## Abstract

As an important metal three-dimensional printing technology, electron beam melting (EBM) is gaining increasing attention due to its huge potential applications in aerospace and biomedical fields. EBM processing of Ti-6Al-4V as well as its microstructure and mechanical properties were extensively investigated. However, it is still lack of quantitative studies regarding its microstructural evolution, indicative of EBM thermal process. Here, we report α′ martensitic transformation and α/β interface evolution in varied printing thicknesses of EBM-printed Ti-6Al-4V block samples by means of atom probe tomography. Quantitative chemical composition analysis suggests a general phase transformation sequence. By increasing in-fill hatched thickness, elemental partitioning ratios arise and β volume fraction is increased. Furthermore, we observe kinetic vanadium segregation and aluminum depletion at interface front and the resultant α/β interface widening phenomenon. It may give rise to an increased α/β lattice mismatch and weakened α/β interfaces, which could account for the degraded strength as printing thickness increases.

Three-dimensional (3D) printing, formally known as additive manufacturing (AM), that can, in principle, create any shape of physical objects by building up successive layers of material from a 3D file, was reported to be ushering the third industrial revolution^1^,^2^. Powder bed based metal 3D printing is one of such newly emerging disruptive manufacturing technologies, and it has been increasingly used in industries which have high value added and low production batch, e.g. orthopaedic implants, dental implants and aerospace components, due to its high manufacturing accuracy. Herein, electron beam melting (EBM) is a representative powder bed based metal 3D printing method, using electron beam as heat source, which is capable of manufacturing complex metallic parts under elevated temperatures and high vacuum^1^,^3^. Ti-6Al-4V is a classical α/β dual phase titanium alloy, and an important engineering material with wide applications^4^. Also, it is the most developed material for EBM processing till now. Fabrication and characterisation of EBM-printed Ti-6Al-4V were extensively conducted within the past 5 years^5^,^6^,^7^,^8^. However, there is still a lack of direct evidence to verify its phase transformation sequence involved in EBM. Of particular interest is the study of martensitic transformation because of its significance to engineer EBM-printed Ti-6Al-4V’s microstructure.

Structural phase transformations in high-angle grain boundaries, i.e. so called planar complexions, are considered to have both fundamental interest and practical importance for polycrystalline materials^9^,^10^. Recently, Kuzmina *et al*.^11^ found constrained austenitic phase at martensitic dislocation cores in a binary Fe-(9 at.%) Mn alloy, which extends the planar complexion concept to the linear case. In the present study, we observed constrained α phase inside heavily faulted and/or twinned α′ martensite in EBM-printed Ti-6Al-4V. In addition, printing thickness dependent microstructure and mechanical properties were lately reported for EBM-printed Ti-6Al-4V^12^. Moreover, α/β interface was found to play a primary role in strengthening EBM-printed Ti-6Al-4V via acting as barriers against dislocation motion^8^. Therefore, an attempt was made to explain the microstructure-property relation from the viewpoint of α/β heterophase interface. Atom probe tomography (APT) is a unique and powerful technique that can locate alloying elements and obtain their compositions quantitatively at atomic scale and in 3D mapping for bulk samples^13^. Thus, APT will be helpful to study multiphase transformation and interphase interface in EBM-printed Ti-6Al-4V. In particular, a quantitative investigation into the microstructural evolution, including phase transformation as well as α/β interface evolution, can provide an in-depth understanding of the complex thermal process of EBM. Furthermore, it will be an important reference for the microstructural study of new materials manufactured by EBM. In this work, four Ti-6Al-4V block samples were 3D printed with printing thicknesses of 1 mm, 5 mm, 10 mm and 20 mm (see Methods). We name them 1 mm, 5 mm, 10 mm and 20 mm samples accordingly. Since they have different printing thicknesses, the in-fill hatching time is supposed to be different and thus their microstructure should differ due to different thermal history.

## Results and Discussion

### Martensitic formation and decomposition

**Figure 1:**
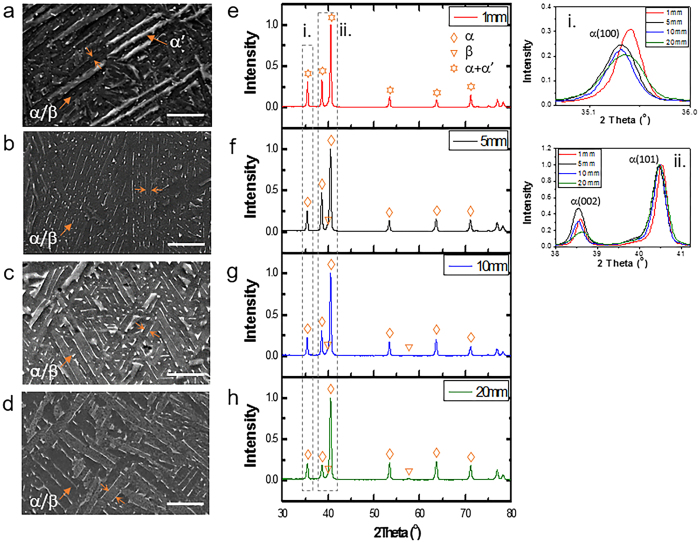
Microstructural differences among the four EBM-printed Ti-6Al-4V samples with varying printing thickness. (**a–d**) Scanning electron microscopy (SEM) images showing microstructure of 1 mm, 5 mm, 10 mm and 20 mm samples, respectively. Scale bar = 5 μm. Martensitic microstructure can only be observed in 1 mm sample. β phase (in white) exhibits a morphology of discrete rod that embedded into continuous α phase (in dark). Distance between two facing arrows denote β phase spacing. Obviously, microstructure becomes coarser with an increase in building thickness. (**e–h**) XRD profiles of 1 mm, 5 mm, 10 mm and 20 mm samples, respectively. Insets (i) and (ii) are enlarged profiles showing α(100), α(002) and α(101) peaks. The peak position shift takes place due to the changes in composition of each phase.

Figure 1a–d show the microstructures of the four samples with different thicknesses. It is evident that β spacing markedly increases with the printing thickness. In addition, we observed a mixed microstructure of α/β and martensite plates in 1 mm sample; while only α/β dual phase microstructure exists in 5 mm, 10 mm and 20 mm samples. X-ray diffraction (XRD) profiles in Fig. 1e–h further verify the phase constitution in each sample. Our previous work shown that the martensite formed in EBM-printed Ti-6Al-4V was of hexagonal type α′^12^. We note that both of α and α′ have hexagonally close-packed (hcp) crystal structure and their lattice parameters are very close to each other. Enlarged XRD profiles in Fig. 1 insets i and ii reveal that the lattice unit cell of α phase is smaller in the 1 mm sample as compared to that in the 5 mm sample, and it tends to decrease with the increase of printing thickness for the three thick samples. β spacings as well as their corresponding Vickers hardness and tensile strength values of the four experimental samples are compiled in Supplementary Table S1. The coarser the microstructure, the lower its hardness and strength. Nonetheless, it should be noted that the highest hardness obtained from the 1 mm sample is not only due to its fine microstructure but also the presence of martensite. Transmission electron microscopy (TEM) results reveals that α′ martensite plates have numerous twin-like and/or stacking faults substructures in 1 mm sample; while limited numbers of twins exist inside α phase in 10 mm sample (see Supplementary Fig. S2).

**Figure 2:**
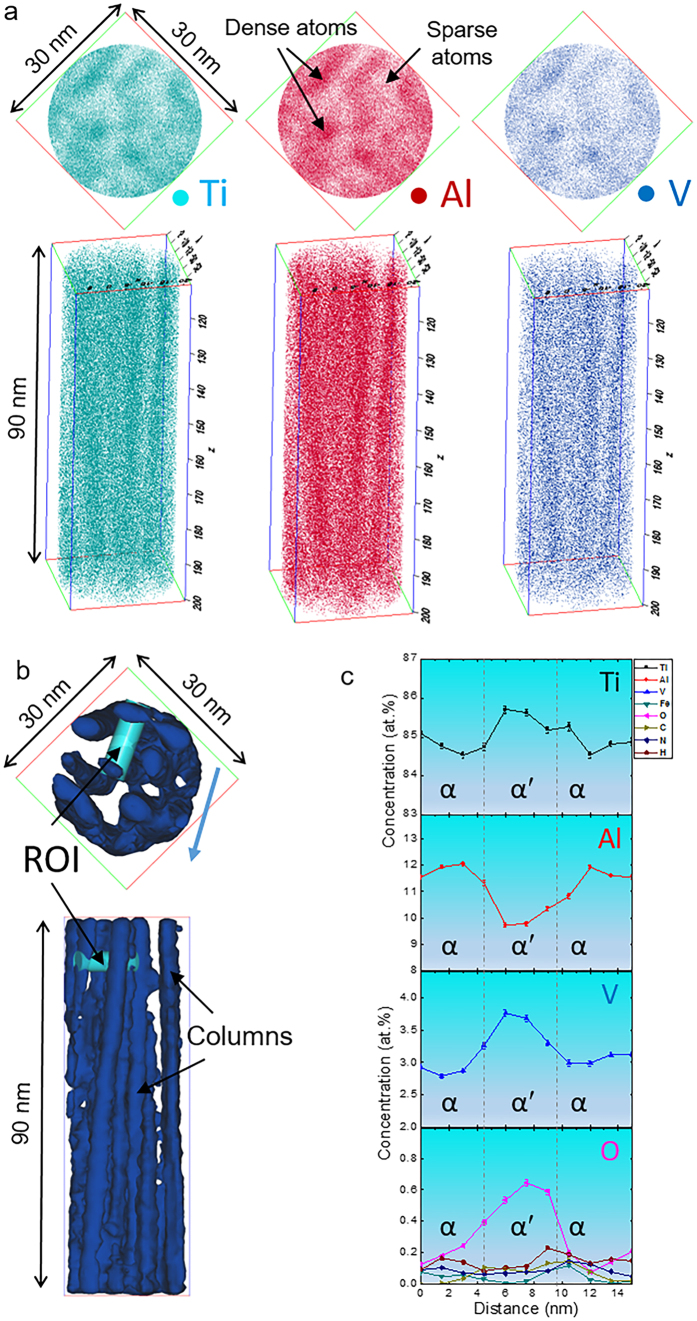
Partial decomposition of α′ martensite in the thin EBM-printed Ti-6Al-4V sample. (**a**) Element distribution of Ti, Al and V from views of transverse and longitudinal directions respectively in a reconstructed volume of 1 mm sample. (**b**) A cylindrical ROI cross over columns that delineated with iso-density surfaces of Ti + Al + V = 20 at. nm^**−**1^. The columns with a higher atomic density were indicated by arrows. (**c**) 1D concentration profiles of all alloying elements in the ROI along the arrow direction indicated in (**b**). As the dense atom regions have very close compositions with initial Ti-6Al-4V ELI (see Supplementary Table 1), they were determined to be martensite. Its crystal structure was determined to be hcp in terms of SAED pattern in Supplementary Fig. 2a. Constrained α phase in α′ martensite is confirmed, indicating martensitic formation and its partial decomposition from nano scale.

Figure 2a displays the elemental distribution of Ti, Al and V in a Φ30 × 90 nm^3^ cylinder that was extracted from an APT reconstructed volume of the 1 mm sample. There are two distinct regions, i.e. dense atom columns and sparse atom matrix. Such a difference in atomic density indicates a difference in phase^14^. In order to better distinguish these two regions, iso-density surfaces of Ti + Al + V = 22 at. nm^**−**1^ were generated. These columns in Fig. 2b seem to be dislocations or stacking faults in terms of their morphology, which is consistent with the image of heavily faulted and/or twinned martensite in Supplementary Fig. S2a. We created a cylindrical region of interest (ROI) that perpendicularly passes through the dense and sparse atom regions as shown in Fig. 2b. 1D concentration profiles in Fig. 2c clearly reveal the varied compositions in these two regions. As compared to Supplementary Table S1, we found that the sparse atom region has very close compositions with initial Ti-6Al-4V ELI. The high-temperature body-centered cubic (bcc) β phase will be decomposed via diffusional, diffusionless or mixed modes upon cooling^15^. Martensitic transformation is the most important type of military transformations, which do not require diffusion for the change in crystal structure to occur. Therefore, the sparse atom matrix should be the martensite that retained the same compositions of high-temperature β phase. With the aid of SAED technique, we confirm that it is α′ martensite. A detailed discussion on the formation of α′ martensite in EBM-printed Ti-6Al-4V can be found elsewhere^8^,^12^. In this work, EBM printing temperature is ~600–650 °C, which is believed to be within M_S_ and M_F_ (start and finish temperature for martensite) of α′^4^. The dense atom columns contain as high as ~12 at.% Al and should be α phase in terms of the Ti-Al-V ternary phase diagram^16^. This α phase should be termed the primary α as it was firstly transformed from β phase during EBM cooling. Thus, β → α′ + α occurred, i.e. the martensitic transformation could not proceed completely since EBM printing temperature is higher than M_F_. Obviously, our results confirm constrained α phase within the heavily faulted and/or twinned α′ martensite. As both stacking faults and twin boundaries are planar defects in crystals, the appearance of constrained α phase can be regarded as planar complexion phenomenon.

**Figure 3:**
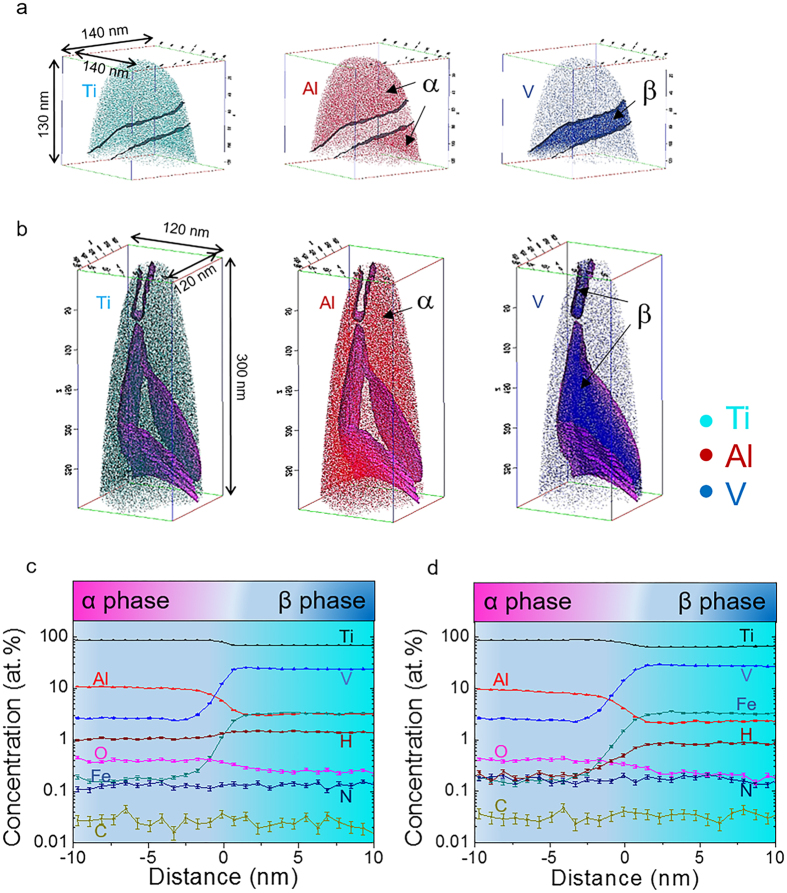
Full decomposition of α′ martensite in the thick EBM-printed Ti-Al-4V samples. (**a,b**) Elemental mappings of Ti, Al and V in 10 mm and 20 mm samples, respectively. 13 at.% and 16 at.% V iso-concentration surfaces (iso-surfaces) were adopted in the APT reconstructed volumes of 10 mm and 20 mm samples, respectively. The values of 13 at.% and 16 at.% were chosen because they approximately enable the solvent elemental concentrations in α and β phases lie midpoint in the proxigrams. α and β phases can be clearly distinguished as the former is rich in Al and the latter rich in V. No martensite was observed, indicative of full martensitic decomposition occurs. (**c,d**) Proxigrams showing elemental partitioning behavior across α/β interfaces in (**a,b**), respectively. Partitioning behavior of each element and quantitative compositions of α and β phases are easily obtained from the proxigrams.

Elemental distribution of Ti, Al and V in APT reconstructed volumes of 10 mm and 20 mm samples are given in Fig. 3a,b. Two phases are present as shown by the difference in composition. Al is α phase stabilizer while V is β phase stabiliser^4^. In addition, the microstructure of EBM-printed Ti-6Al-4V consists of fine β rods embedded into continuous α phase. Therefore, α and β phases are easily distinguished. Figure 3c,d present the two proxigrams that were generated on the base of 13 at.% and 16 at.% V iso-concentration surfaces (iso-surfaces) in the APT reconstructed volumes of 10 mm and 20 mm samples, respectively. Both proxigrams reveal that Ti, Al and O preferentially partition to α phase while V, Fe and H more likely enter β phase. Moreover, no evident partitioning trend was observed for C and N. Table 1 reveals that Al and V are the most heavily partitioned alloying elements. Moreover, the partitioning ratios were markedly increased in 20 mm sample when compared to 10 mm sample.

Finite element method (FEM) simulation results shown that both cooling rate and thermal profile involved in EBM processing of these four samples were favourable to martensitic formation^12^. However, no martensite was observed in thick samples (5 mm, 10 mm, and 20 mm thick), indicative of that α′ martensite is unstable within the printing temperature. It was reported that decomposition of martensite would take place via nucleation and growth or spinodal modes when it was subject to tempering^17^. In this work, we observed the fully decomposed α/β dual phases from SEM (Fig. 1b–d), TEM (Supplementary Fig. S2b) and APT (Fig. 3a,b) in 5 mm, 10 mm and 20 mm samples. Moreover, partial decomposition of α′ martensite could be seen in terms of Figs 1a,e and 2. It is well known that “up-hill” diffusion and diffuse interface between precipitate and matrix are the main characteristics during the early stage of spinodal decomposition^18^. Our results reveal that α′ martensite in EBM-printed Ti-6Al-4V may decompose into α/β through the nucleation and growth mode due to the “down-hill” diffusion and sharp interface between α′/α. Furthermore, no satellite spot was detected in SAED patterns of α′ martensite. Apart from the printing temperature, another key factor that could accelerate α′ martensitic decomposition might be attributed to the heat affected zone that originates from the previous molten layer.

**Figure 4:**
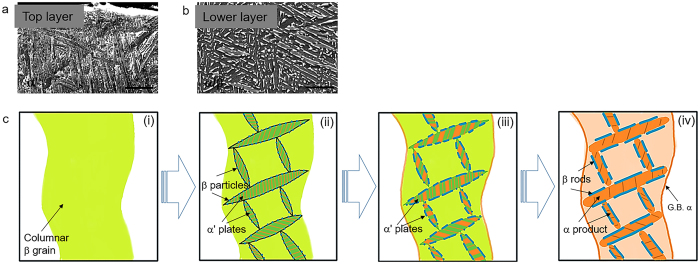
General microstructural evolution of EBM-printed Ti-6Al-4V. (**a,b**) SEM images showing martensitic microstructure at the very top area and fully decomposed α/β microstructure at the lower area of 10 mm sample. It demonstrates that martensite will form during EBM printing of Ti-6Al-4V regardless of its printing thickness. Scale bar = 5 μm. (**c**) Schematic illustration of microstructural evolution (prior β and α′ are in cyan, α in orange and β in blue), i.e. martensitic formation and decomposition, involved in EBM-built Ti-6Al-4V. Complexions occur at stacking faults and/or twin boundaries in α′ martensite. α phase will continuously grow at the expense of α′ and β rods appear due to the ejecting vanadium from growing α. At last, fully decomposed microstructure with β rods embedded into continuous α phase forms.

Figure 4a,b show the partially decomposed martensitic microstructure at the very top region and the fully decomposed α/β microstructure at a lower region in 10 mm sample respectively, which strongly suggest the martensitic formation regardless of printing thickness. Metal 3D printing is a layerwise melting process^1^. The previously deposited layer was heat treated and then cooled down repeatedly several times when the successive layers were melted, resulting in time-dependent, cyclic temperature profiles within the part being printed, until its local temperature became stable at a soak temperature. The thermal cycling duration is highly dependent on the processing parameters and the printing geometries. If the soak temperature is sufficiently high to induce phase transformation for the printing material, we may observe obvious microstructural difference between the very top region and the remaining bulk region. For EBM processing of Ti-6Al-4V, the soak temperature of ~600–650 °C is sufficient to result in the decomposition of α′ martensite. Therefore, we observe the typical α/β duplex microstructure in the Ti-6Al-4V bulk while martensitic microstructure appears at the very top region. It is obviously an indication of the thermal cycling process. By contrast, if the soak temperature is close to ambient environment, e.g. selective laser melting (SLM), martensitic phase will be maintained without significant decomposition^19^.

Figure 4c schematically illustrates the α′ martensitic formation and decomposition during EBM process. Firstly, wavy columnar β grains might transiently form and then transformed into heavily faulted and/or twined α′ plates as well as some retained β phase (i → ii). During the decomposition of α′ into α, vanadium is constantly rejected from the forming α phase and diffuse along accommodation twin boundaries and/or stacking faults towards the boundaries between adjacent α′ martensite plates. The intergrowth of α within α ′plates could be taken into account as planar defects driven growth, which can refer to the author’s previous work^20^. Discrete β particles will first form along α′ martensite plate boundaries and then continuously grow at expense of vanadium (ii → iii). Accordingly, these grown discrete β particles may be connected and β rods would eventually form at the fully decomposed α plate boundaries (iii → iv). To differentiate with the primary α that formed during rapid cooling, the α phase appeared after decomposition should be named secondary α. Thus, the complete phase transformation path that involved in EBM processing of Ti-6Al-4V should be as follows: *S*_*(powder)*_ → *L*_*(melt)*_ → *β*_*prior*_ → *α*′ + *α*_*primary*_ + *β*_*retained*_ → *α*_*secondary*_ + *β*. We derived this phase transformation path from extensive microstructural observation and quantitative chemical and structural investigation on the Ti-6Al-4V builds with varying thickness, which represents the different stages of EBM printing. Meanwhile, it correlates the relationship between EBM thermal cycling and Ti-Al-V phase diagram as well.

### α/β interface evolution

As the microstructures of 10 mm and 20 mm samples were only comprised of α and β phases, it obeys *C*_*0*_ = *C*_*α*_∙*V*_*f*_* (α)* + *C*_*β*_∙*V*_*f*_* (β)*, where *C*_*0*_ is the overall composition of Ti-6Al-4V ELI; *C*_*α*_ and *C*_*β*_ are the compositions of α and β phases; *V*_*f*_* (α)* and *V*_*f*_* (β)* are the volume fractions of α and β phases, respectively. Then *V*_*f*_* (β)* can be derived from the following formula:


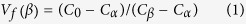


**Figure 5:**
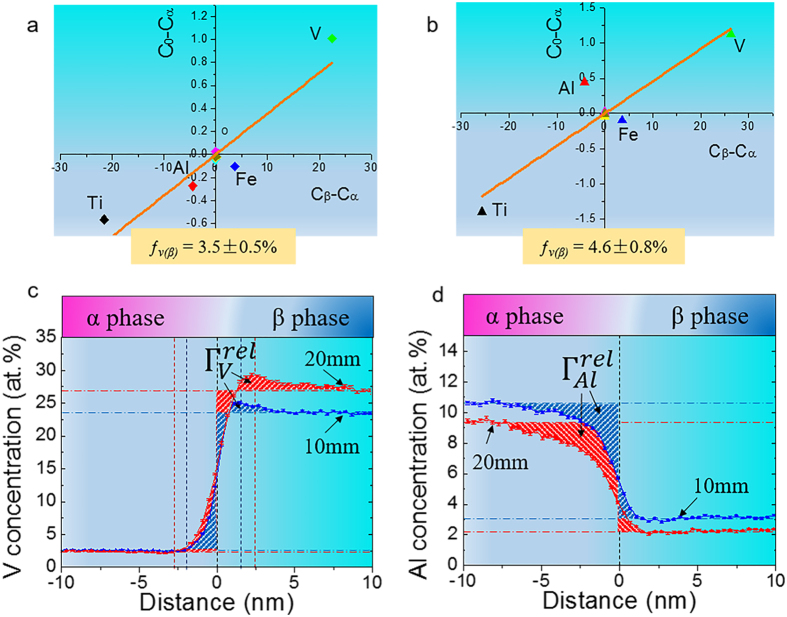
β phase volume fraction change and α/β interface characteristics. (**a,b**) (C_0_-C_α_)-(C_β_-C_α_) plots for 10 mm and 20 mm samples, respectively. β volume fraction is found to be increased with building thickness. As β (bcc) is softer than α (hcp), the significant increase in β volume fraction will definitely reduce material’s hardness and strength. (**c**) Vanadium concentration profiles showing kinetic vanadium segregation (hatched region) at interface front of β phase in 10 mm and 20 mm samples. (**d**) Aluminum concentration profiles showing kinetic aluminum depletion (hatched region) at interface front of α phase in 10 mm and 20 mm samples. α/β interface was found to be widened in 20 mm sample due to vanadium segregation and aluminum depletion.

The *(C*_*0*_*-C*_*α*_*)–(C*_*β*_*-C*_*α*_) graphs were plotted and the slope of the fit line is approximately equal to the volume fraction of β phase. Hence, the β volume fraction in 10 mm and 20 mm samples can be determined to be 3.5 ± 0.5% and 4.6 ± 0.8% from Fig. 5a,b. The enhanced β volume fraction with the increase in printing thickness is thought to be related with the larger thermal mass of thick sample. A large thermal mass enables EBM builds to stay at a high temperature for some time, which has been verified by FEM simulations^12^. The EBM printing temperature of ~600–650 °C induces that the main microstructural evolution takes place at α + β two phase field in terms of Ti-6Al-x%V pseudo phase diagram. Therefore, a rising temperature would result in an increased volume fraction of β phase. It is worth noting that the β stabilizer of Fe that potentially diffused from the stainless steel start plate could also significantly increase the volume fraction of β phase particularly for the base of an EBM-printed Ti-6Al-4V part. However, its influence is avoided in this work as the specimens for APT detection were extracted at a printing height of ~20 mm away from the base. As β-Ti (bcc) is considerably softer than α-Ti (hcp)^21^, the increase in β volume fraction would give rise to material strength deterioration from a macroscopic view.

As-printed Ti-6Al-4V by EBM could have superior tensile properties as compared to as-cast form largely owing to its fine Widmanstätten α/β microstructure. The Hall-Petch relation^22^ can help to understand why the hardness or strength of EBM-printed Ti-6Al-4V samples will be decreased with increasing printing thickness; indeed the finer microstructure will lead to a higher strength. We can presume that the higher strength obtained from the finer β spacing is due to more α/β interfaces that the microstructure contains. It was observed that dislocations were always first emitted from α/β interfaces in Ti-6Al-4V and plastic deformation started with dislocations emitted from there^23^. Moreover, α/β interface is believed to play the primary role in strengthening EBM-printed Ti-6Al-4V^8^. It thus becomes very necessary to study the characteristics of α/β interface in order to explain the printing thickness dependent properties from a more microscopic viewpoint.

We quantify elemental segregation behavior at α/β interface utilising the relative Gibbsian interfacial excess (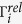), which is defined by the excess number of solute atoms *i* (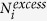) per unit area (*A*) for an arbitrary interface, i.e.:^14^


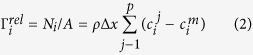


where *ρ* is the atomic density (theoretically, 61.0 at. nm^**−**3^ for β and 57.6 at. nm^**−**3^ for α in this work), Δ*x* is the distance between the *p*-layers in the proxigram, 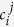 is the concentration of the *j*th atom, and 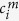 is the average concentration of element *i* in the matrix. Therefore, we can estimate the interfacial excess values of vanadium and aluminum as the hatched areas in terms of the proxigrams displayed in Fig. 5c,d. The chemistry (i.e. chemical composition), species and anisotropy of matrix-precipitate interfaces may significantly impact interfacial energy via solute atom segregation for multicomponent alloys^24^. In terms of Gibbs adsorption theorem at constant temperature and pressure, the coefficient of reduction of total interfacial energy *E*_*tot*_ due to segregation of component *i* with concentration *c*_*i*_ at the interface is given by^24^,^25^,^26^


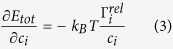


Here, if we assume a linear dependence of *E*_*tot*_ on solute segregation, the total reduction of interfacial energy ∂*E*_*tot*_ can be determined by 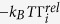. Boltzmann constant *k*_*B*_ ≈ 1.38e-23 J K^**−**1^ and T = 923 K (i.e. EBM printing temperature) were adopted. Table 2 compiles the 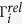 values of vanadium and aluminum as well as the resultant reduction in *E*_*tot*_ for both of 10 mm and 20 mm samples. Due to its low diffusivity in β, vanadium concentration will form a “bump” in β phase adjacent to α/β interface (see Fig. 5c). As the Gibbs dividing plane nearly locates at the middle of β concentration profile, the Gibbsian interfacial excess of vanadium can be only considered as the hatched “bump” area. Apparently, more vanadium segregates at the vicinity of α/β interface in the thicker sample. Of particular note is that aluminum has a strong tendency toward depletion at the interface with negative excess values. Moreover, more aluminum was depleted at α/β interface with an increase in printing thickness. Both vanadium segregation and aluminum depletion were found to significantly influence the α/β heterophase interfacial energy and the latter is more influential. In general, the total interfacial energy seems slightly reduced from 10 mm to 20 mm samples because of solutes’ interaction with interface. The reduction of total interfacial energy is supposed to be the thermodynamic driving force for β phase coarsening.

The Burgers relationship exists between α phase and β phase, i.e. (0001)_α_//(110)_β_ and [11
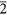
0]_α_//[111]_β_, and α/β interface is semi-coherent^27^. Approximately, the total interfacial energy (*E*_*tot*_) of a semi-coherent interface consists of chemical interaction energy *E*_*chem*_ (as for a fully coherent interface) and elastic strain energy *E*_*el*_ (as the extra energy due to structural distortion caused by mismatch dislocations), i.e. *E*_*tot*_ = *E*_*chem*_ + *E*_*el*_^18^. Elmer *et al*.^28^ conducted an *in-situ* study of the lattice thermal expansion for α → β transformation on heating. We can know that α phase has a larger unit cell volume than β phase from room temperature to β transus temperature^28^. As mentioned above, both of the partitioning ratios of aluminum and vanadium were increased with increasing printing thickness. It demonstrates that more aluminum partitions to α, in the meanwhile more vanadium enters β phase, for 20 mm sample when compared to 10 mm sample. By increasing vanadium content, the lattice constant of β phase will be decreased^28^. Likewise, the lattice constants of α phase will also be reduced with increasing aluminum content due to their smaller atomic radius in comparison with titanium. This explains why α lattice becomes increasingly smaller in printing thickness from 5 mm to 20 mm (see Fig. 1 insets i and ii). Furthermore, α lattice is smaller in 1 mm sample because it contains a very high level of aluminum. The severer vanadium segregation at the interface front of β phase is believed to further reduce β lattice constant. By contrast, more aluminum depletion at the interface front of α phase is supposed to increase α lattice constants. As a result, it gives rise to a larger lattice mismatch (δ) at α/β interfaces in 20 mm sample. For small values of δ (usually <0.25) structural contribution to interfacial energy is approximately proportional to lattice mismatch at interface, i.e. *E*_*el*_ ∝ *δ*^18^. Thus, the interfacial elastic energy of 20 mm sample is higher than that of 10 mm sample. It implies that elastic interaction could account for the interfacial segregation of vanadium and depletion of aluminum as displayed in Table 2.

In addition, Table 2 also shows that α/β interface width of 20 mm sample, which was determined by vanadium concentration profile, becomes wider as compared to that of 10 mm sample. Although it is possible to obtain a broad interface owing to the field evaporation trajectory effect based on proxigrams^25^, α/β interface of 20 mm sample is believed to be widened to some extent due to the more enrichment of vanadium and depletion of aluminum at its interface front. The larger lattice mismatch will enable dislocations to be more easily generated at α/β interfaces once deformation begins. On the other hand, the widening of interface width will further weaken α/β interface strength. Therefore, mechanical properties, e.g. hardness and tensile strength, will degrade with the increase of printing thickness for EBM-printed Ti-6Al-4V. One may say that the change of α/β interface resulted from thermal effect could be of importance for the variation of mechanical properties for EBM-printed Ti-6Al-4V.

Overall, this work demonstrates that α′ martensite is firstly formed in EBM processing of Ti-6Al-4V regardless of printing geometries. Unlike other powder-based metal 3D printing methods, the martensitic phase is then decomposed into α/β microstructure to different extent relying on the subsequent thermal cycling history. We reveal the formation and decomposition of α′-Ti martensite from quantitative structural and chemical information, with a view to enrich the knowledge on martensite in titanium alloys. In terms of the general phase transformation path as suggested in this work, it is possible to further modify the microstructure and optimize its compressive mechanical properties for EBM-printed Ti-6Al-4V parts by varying printing temperature. We can reduce the average beam current of EBM process to achieve a lower printing temperature, for example, and then a mixed microstructure of (α′ + α/β) that is strong and tough could be obtained. The martensitic formation and decomposition observed in this work also confirms the EBM thermal cycling, which could aid to better understand the microstructure of other materials printed by EBM. In addition, quantitative microstructural characterization helps us understand the deteriorating strength with the increase in in-fill hatching thickness from a macroscopic view, namely, that is due to the increasing β spacing and β volume fraction. Moreover, we quantitatively examine the elemental partitioning and segregation behavior at the α/β heterophase interface. The increasing α/β lattice mismatch and α/β interface width are believed to account for the strength degradation from the microscopic view.

## Methods

### Sample preparation

We use an Arcam A2XX (Arcam AB, Sweden) EBM machine (its schematic is given in Supplementary Fig. S1a) to 3D print Ti-6Al-4V samples, using Ti-6Al-4V ELI (Grade 23) powder supplied by Arcam AB. The powder size distribution ranges from 45 to 106 μm (see Supplementary Fig. S1b). Supplementary Table S2 lists the nominal composition of as-supplied powders. Recycling of non-melted and/or sintered powder was achieved via the powder recovery system (PRS) and a vibrating sieve (mesh size ≤150 μm). Standard Ti-6Al-4V printing parameters provided by Arcam were used to print all samples. The detailed EBM fabrication process can be found elsewhere^29^. In order to investigate the microstructural evolution of EBM-printed parts with different thermal process, four types of block samples with the same XZ (100 mm × 30 mm) but varying thicknesses of 1 mm, 5 mm, 10 mm and 20 mm were printed using the standard processing parameters by Arcam (see Supplementary Fig. S1c). They were printed directly onto the preheated start plate by selective melting layers of 50 μm under a controlled vacuum in a temperature range of 600–650 °C. The entire printing process was kept under vacuum at ~2e-3 mBar, controlled by using high-purity helium as regulating gas in order to prevent powder charging. All the specimens for microstructural characterization and mechanical testing were extracted at the same sample height of ~20 mm.

### Characterisation techniques

We use scanning electron microscopy (SEM; JEOL JMS-6700F; 20 kV), X-ray diffraction (XRD; PANalytical Empyrean; Cu Kα; step size of 0.013°), transmission electron microscopy (TEM; JEOL-2010; 200 kV), and atom probe tomography (APT; LEAP 3000X HR; 200 kHz; 40 K; 0.9 nJ pluse^**−**1^) to examine the microstructure of as-EBM-print Ti-6Al-4V samples. SEM samples were etched in Kroll’s reagent (1–3% HF, 2–6% HNO_3_, and 91–97% H_2_O) for 10 s. TEM samples were prepared using the following procedures: thin sample sections were ground to ~100 μm and then manually ground to ~50 μm with silicon carbide paper under the protection of water; 3 mm (in diameter) TEM discs were punched out from the 50 μm thick sections; and the disc was dimpled on both sides and then Ion milled within 4–8° with varied voltages from 3.5 to 4.5 V. APT specimens were prepared by focused ion beam (FIB) on a FEI Helios dual-beam via the lift-out technique and the micro-tips were prepared using the annular milling method^30^ to obtain an end radius of ~50 nm. APT specimens were analysed at 40 K and a gauge pressure <2e-11 Torr. Pulses of green laser light (532 nm wavelength) were applied at a 200 kHz repetition rate with an energy of 0.9 nJ pulse^**−**1^, yielding an evaporation rate of 0.30%. We analyze APT data using IVAS^®^ 3.6.6 software and obtain compositional information employing the proximity histogram (proxigram) methodology^31^. Errors bars were plotted in each compositional profile. The error bars were calculated using 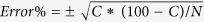, where *C* is the composition value in at.% and *N* is the number of atoms in the distance step over which these values were being averaged^32^.

### Mechanical testing

We conduct tensile tests on an Instron Static Tester (series 5569) using subsize specimens with a gauge dimension of 25 mm × 6 mm × 6 mm according to ASTM E8 standard at a strain rate of 3.33e-4 s^**−**1^. Yield strength (YS, 0.2% offset method), elongation at break (% EL) and ultimate tensile strength (UTS) were measured from the engineering stress-strain curves. Vickers microhardness (HV) (100 g f, 15 s hold) measurements were performed on the metallographic samples using a Future Tech FM-300e microhardness tester.

## Additional Information

**How to cite this article**: Tan, X.P. *et al*. Revealing martensitic transformation and α/β interface evolution in electron beam melting three-dimensional-printed Ti-6Al-4V. *Sci. Rep.* **6**, 26039; doi: 10.1038/srep26039 (2016).

## Supplementary Material

Supplementary Information

## Figures and Tables

**Table 1:** Compositions (in at.%) of α and β phases in 10 mm and 20 mm samples obtained from APT data as well as the partitioning ratio of each element.

	**Sample**	**Ti**	**σ (Ti)**	**Al**	**σ (Al)**	**V**	**σ (V)**	**Fe**	**σ (Fe)**	**O**	**σ (O)**	**C**	**σ (C)**	**N**	**σ (N)**	**H**	**σ (H)**
α	10 mm	85.15	0.13	10.49	0.12	2.65	0.06	0.16	0.02	0.41	0.02	0.03	0.01	0.12	0.01	0.99	0.04
	20 mm	87.02	0.14	9.41	0.12	2.57	0.07	0.16	0.02	0.42	0.03	0.03	0.01	0.18	0.02	0.20	0.02
β	10 mm	68.22	0.18	3.13	0.07	23.68	0.16	3.28	0.07	0.24	0.02	0.03	0.01	0.13	0.01	1.29	0.04
	20 mm	65.66	0.21	2.32	0.07	27.43	0.20	3.34	0.08	0.20	0.02	0.03	0.01	0.16	0.02	0.86	0.04
k or k′	10 mm	1.2		3.4		8.9		20.5		1.7		1.0		1.1		1.3	
	20 mm	1.3		4.1		10.7		20.9		2.1		1.0		1.1		4.3	

The partitioning ratio *k* (for α-partitioning elements) or *k*′ (for β-partitioning elements) were defined as or *k* = *C*_*α*_/*C*_*β*_ or *k*′ = *C*_*β*_/*C*_*α*_, respectively. The larger than unity for *k* or *k*′, the severer partitioning for the corresponding element.

**Table 2:** Relative Gibbsian interfacial excess ( 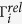 ) as well as the change in interfacial energy(Δ*E*_*tot*_).

**Sample**	**Solute**	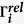 **(at. nm**^−**2**^)	***∆E***_***tot***_**(mJ m**^**−2**^)	***w***_***α/β***_ **(nm)**
10 mm (923 K)	V	2.2 ± 0.1	−28.0	2.2 ± 0.1
	Al	−4.3 ± 0.3	+54.8	–
20 mm (923 K)	V	5.3 ± 0.4	−67.5	5.3 ± 0.4
	Al	−7.0 ± 0.5	+89.2	–
