# Supplementary material for: Revealing martensitic transformation and α/β interface evolution in electron beam melting three-dimensional-printed Ti-6Al-4V
Source: Sci Rep. 2016 May 17;6:26039. doi: 10.1038/srep26039 (PMC4869034; doi:10.1038/srep26039)
Supplement: Supplementary Information [file srep26039-s1.pdf]

Revealing martensitic transformation and α/β interface evolution in electron beam melting three-dimensional-printed Ti-6Al-4V

Xipeng Tan[1*], Yihong Kok[1], Wei Quan Toh[1], Yu Jun Tan[1], Marion Descoins[2], Dominique Mangelinck[2], Shu Beng Tor[1], Kah Fai Leong[1], Chee Kai Chua[1]

[1]Singapore Centre for 3D Printing, School of Mechanical & Aerospace Engineering, Nanyang Technological University, 50 Nanyang Avenue, Singapore 639798

[2]IM2NP, UMR 7334 CNRS, Université Aix-Marseille, 13397 Marseille Cedex 20, France

[*]Corresponding author. E-mail: xptan1985@gmail.com & xptan@ntu.edu.sg

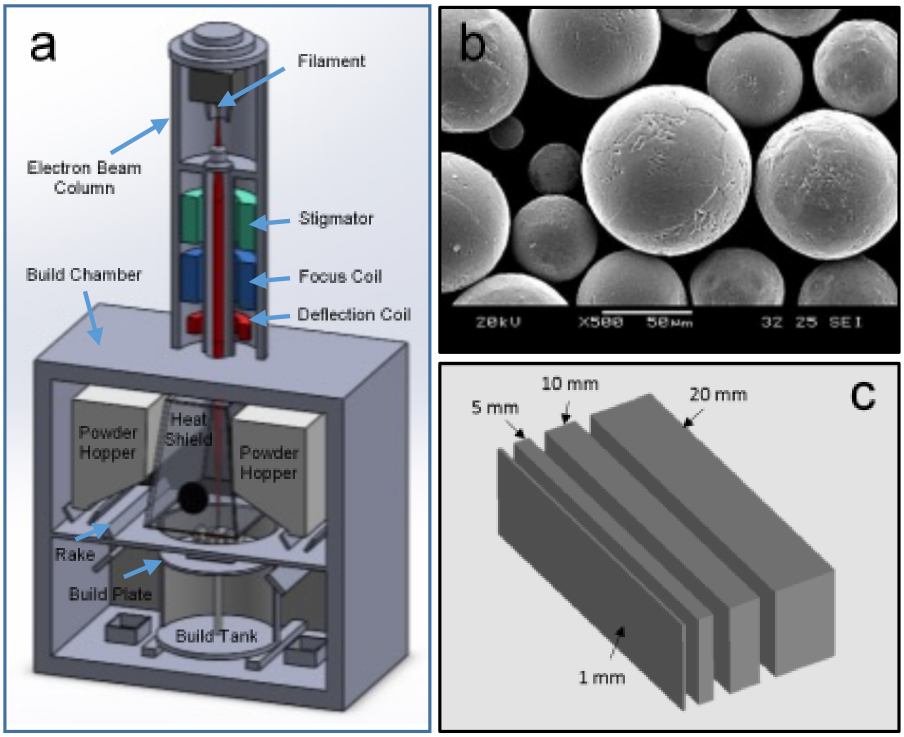

Supplementary Fig. S1. (Color online) (a) Schematic of the EBM system. (b) A SEM image showing Ti-6Al-4V ELI powder. (c) Schematic illustration of four EBM-built block samples with build thicknesses of 1 mm, 5 mm, 10 mm and 20 mm.

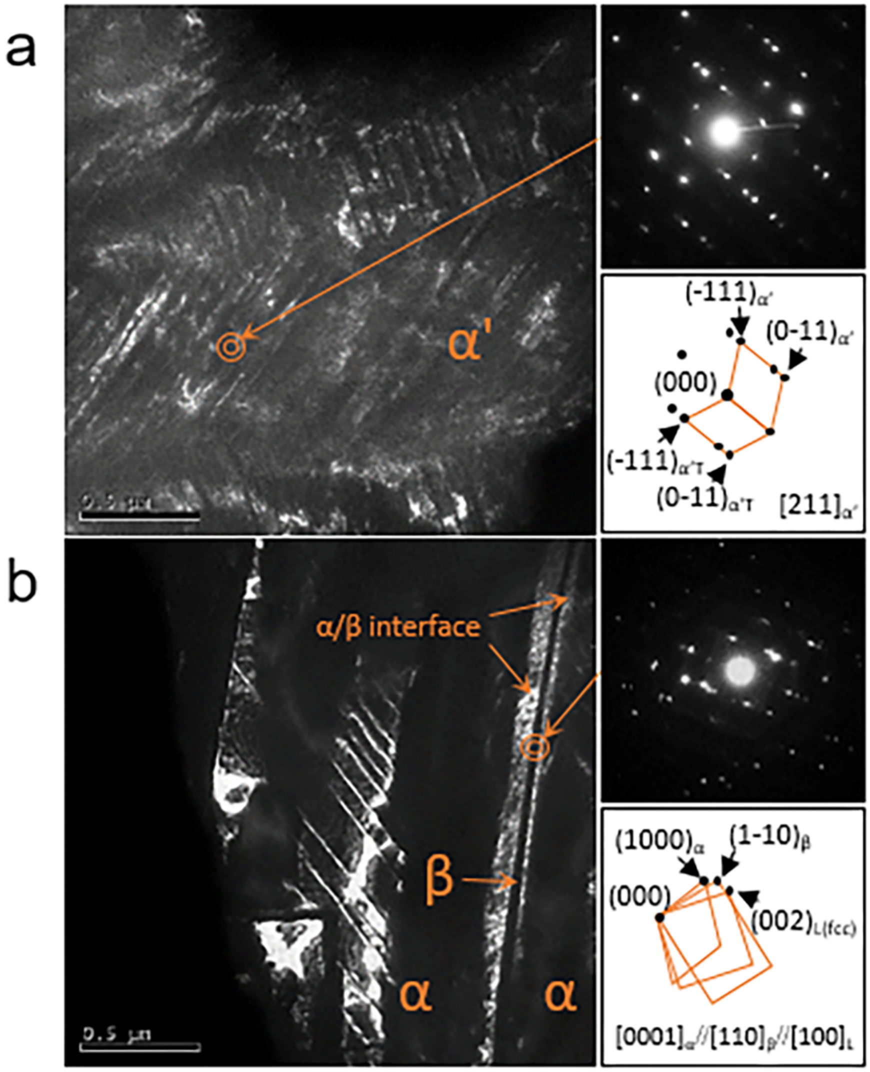

Supplementary Fig. S2. (a) and (b) Dark field TEM images showing microstructure of 1mm and 10mm samples, respectively. Inserts reveals α′ martensite in 1mm sample and the α/L/β phases in 10mm sample. Scale bar = 0.5 µm. It reveals α' martensite plates having numerous twin-like and/or stacking faults substructures in 1mm sample. The orientation of these parallel substructures is shown to be different in each martensite plate. Selected area electron diffraction (SAED) analysis identifies these α' accommodation twins as $\{1\bar{2}10\}$ twins. Moreover, no obvious boundary exists between adjacent α' plates, which may suggest that a diffusionless process indeed take place during their formation. Many twins can be seen inside α phase. Of particular note is the appearance of obvious α/β interface layers. This interface phase was determined as a face-centered cubic (fcc) *L* phase in this work. Our previous work shown that *L* phase had the same composition with α phase and its formation was most likely due to the lattice mismatch between α and β phases via a shear mechanism.

Supplementary Table S1. The β phase spacing as well as corresponding Vickers hardness, yield strength and ultimate tensile strength of the four experimental samples.

Sample	β spacing (μm)	Vickers Hardness (HV)	Yield strength (0.2% offset) (MPa)	Ultimate tensile strength (MPa)
1mm	0.283±0.068	396.8±12.0	—	—
5mm	0.463±0.097	374.7±12.5	—	—
10mm	0.610±0.105	361.1±9.6	829.3±8.2	946.2±8.6
20mm	0.813±0.185	344.2±12.6	799.0±10.7	926.5±4.6

Supplementary Table 2. Nominal composition of pre-alloyed Ti-6Al-4V ELI powder.

	Ti	Al	V	Fe	C	N	O	H
wt.%	Bal.	6	4	0.1	0.03	0.01	0.1	0.003
at.%	Bal.	10.15	3.59	0.08	0.11	0.03	0.29	0.14
